# Pleural fluid soluble triggering receptor expressed on myeloid cells-1 as a marker of bacterial infection: a meta-analysis

**DOI:** 10.1186/1471-2334-11-280

**Published:** 2011-10-20

**Authors:** Hanssa Summah, Li-Li Tao, Ying-Gang Zhu, Hong-Ni Jiang, Jie-Ming Qu

**Affiliations:** 1 Department of Pulmonary Medicine, Huadong Hospital, Shanghai Medical College, Fudan University, Shanghai, People's Republic of China; 2 Department of Pulmonary Medicine, Zhongshan Hospital, Shanghai Medical College, Fudan University, Shanghai, People's Republic of China; 3 Department of Pulmonary Medicine, Huadong Hospital, Fudan University School of Medicine, Shanghai, People's Republic of China

## Abstract

**Background:**

Pleural infection is a common clinical problem. Its successful treatment depends on rapid diagnosis and early initiation of antibiotics. The measurement of soluble triggering receptor expressed in myeloid cells-1 (sTREM-1) level in pleural effusions has proven to be a valuable diagnostic tool for differentiating bacterial effusions from effusions of other etiologies. Herein, we performed a meta-analysis to assess the accuracy of pleural fluid sTREM-1 in the diagnosis of bacterial infection.

**Methods:**

We searched Web of Knowledge and Medline from 1990 through March 2011 for studies reporting diagnostic accuracy data regarding the use of sTREM-1 in the diagnosis of bacterial pleural effusions. Pooled sensitivity and specificity and summary measures of accuracy and Q* were calculated.

**Results:**

Overall, the sensitivity of sTREM-1was 78% (95% CI: 72%-83%); the specificity was 84% (95% CI: 80%-87%); the positive likelihood ratio was 6.0 (95% CI: 3.3-10.7); and the negative likelihood ratio was 0.22 (95% CI: 0.12-0.40). The area under the SROC curve for sTREM-1 was 0.92. Statistical heterogeneity and inconsistency were found for sensitivity (p = 0.015, χ^2 ^= 15.73, I^2 ^= 61.9%), specificity (p = 0.000, χ^2 ^= 29.90, I^2 ^= 79.9%), positive likelihood ratio (p = 0.000, χ^2 ^= 33.09, I^2 ^= 81.9%), negative likelihood ratio (p = 0.008, χ^2 ^= 17.25, I^2 ^= 65.2%), and diagnostic odds ratio (p = 0.000, χ^2 ^= 28.49, I^2 ^= 78.9%). A meta-regression analysis performed showed that the Quality Assessment of Diagnostic Accuracy Studies score (p = 0.3245; RDOR, 4.34; 95% CI, 0.11 to 164.01), the Standards for Reporting of Diagnostic Accuracy score (p = 0.3331; RDOR, 1.70; 95% CI, 0.44 to 6.52), lack of blinding (p = 0.7439; RDOR, 0.60; 95% CI, 0.01 to 33.80), and whether the studies were prospective or retrospective studies (p = 0.2068; RDOR, 7.44; 95% CI, 0.18 to 301.17) did not affect the test accuracy. A funnel plot for publication bias suggested a remarkable trend of publication bias.

**Conclusions:**

Our findings suggest that sTREM-1 has a good diagnostic accuracy and may provide a useful adjunctive tool for the diagnosis of bacterial pleural effusions. However, further studies are needed in order to identify any differences in the diagnostic performance of sTREM-1 of parapneumonic effusions and empyemas.

## Background

Pleural infection (parapneumonic effusion and empyema) or bacterial pleural effusion is a common clinical problem. Its successful treatment depends on rapid diagnosis and early intiation of antibiotics. Delay in diagnosis results in substantial delay in the commencement of treatment and may contribute to the high mortality of this infection. Treatment of all patients with suspected pleural effusion with antibiotics while awaiting for microbiological results is not a good option since this practice increases antibiotic resistance. It is surprising how, in many cases, even the diagnosis and differential diagnosis of parapneumonic effusions poses great problem. Biochemical parameters are often non-specific and Gram stain has a low sensitivity. Pleural fluid cultures, even though being specific, may take days to reveal a positive culture and in 30% to 35% of cases, the organism fails to be cultured [1].

The triggering receptor expressed in myeloid cells-1 (TREM-1) belongs to the immunoglobin superfamily and is involved in inflammatory response [2,3]. TREM-1 exists in both a membranous and a soluble form (soluble triggering receptor expressed on myeloid cells-1; sTREM-1) [4]. TREM-1 is shed by the membrane of activated phagocytes after exposure to bacteria and fungi and, its soluble form, sTREM-1 can be detected in body fluids [5,6]. The measurement of sTREM-1 level in pleural effusions has proven to be a valuable diagnostic tool for differentiating bacterial effusions from effusions of other etiologies [7].

Up to now, no meta-analysis has been undertaken to evaluate the accuracy of pleural fluid sTREM-1 in the diagnosis of pleural effusions. We therefore conducted a meta-analysis of the published literature to assess the accuracy of pleural fluid sTREM-1 for the diagnosis of pleural infection.

## Methods

### Study eligibility

Studies were considered eligible for inclusion in the meta-analysis if they fulfilled the following criteria: original publication; study population included human subjects only; sensitivity and specificity of pleural fluid sTREM-1 for the detection of bacterial infection in pleural effusions could be calculated for patients with proven bacterial effusions.

### Literature search

Literature search was carried out using electronic databases Web of Knowledge (1990 to March 2011) and Medline (1990 to March 2011), with the databases being last assessed on 28 March 2011. We used the terms "sTREM-1", "soluble triggering receptor expressed on myeloid cells-1", "parapneumonic effusion", "empyema", "pleural fluid", and "pleural effusion", whereas the syntax used for Medline searches was (("Pleural Effusion"[Mesh]) OR "Empyema, Pleural"[Mesh]) AND " soluble triggering receptor expressed on myeloid cells 1 protein, human". The search was restricted to human subjects. Studies published only in abstract form were excluded due to the fact that these studies had not undergone peer-review and the inclusion of these studies might introduce bias into the meta-analysis. Case reports, review articles, and textbook chapters were also excluded. The reference lists of all articles reviewed were also searched for eligible studies.

### Study selection and data extraction

Studies were included if they reported the sensitivity and specificity of pleural fluid sTREM-1 in the diagnosis of parapneumonic effusions and/or empyemas. Two authors (HS and LLT) independently reviewed the abstracts of all the studies generated by the computerized search of Web of Knowledge and Medline. The full text articles of all eligible abstracts were reviewed by the two authors. Any discrepancies were resolved by discussion with a third author to reach a final consensus. The data extracted included: (1) publication details including year of publication, title, name of the first author; (2) type of study design (prospective/retrospective); (3) study population; (4) sample size; (5) type of bacterial pleural effusions (parapneumonic effusions and/or empyema); (6) blinding of investigators to results; (7) prevalence of bacterial effusions; (8) assay method; (9) means of diagnosis of bacterial effusions; (10) any cut-off value reported; (11) true positive, true negative, false positive and false negative results; (12) any presence of bias that may have influenced results including incorporation bias (i.e., using pleural fluid sTREM-1 as part of the diagnostic criteria for bacterial effusions).

### Study quality assessment

We used the Quality Assessment Tool for Diagnostic Accuracy Studies (QUADAS tool) [8] and the Standards for Reporting of Diagnostic Accuracy (STARD) checklist [9] for the reporting of studies of diagnostic accuracy for the assessment of the methodological quality of the included studies.

### Data analysis

We performed a diagnostic test accuracy meta-analysis in order to determine the overall sensitivity and specificity of pleural fluid sTREM-1 in the diagnosis of bacterial effusions. Specifically, we used a bivariate random effects model in order to calculate the pooled sensitivity and specificity, pooled positive and negative likelihood ratio [10], as well as the diagnostic odds ratio, along with the respective 95% confidence intervals (CI). We also constructed the summary receiver operating characteristics curve (SROC) [11] and we calculated the respective area under the curve (AUC). We also calculated Q*, where sensitivity = specificity on the summary ROC curve, corresponding to the upper left-most point on the SROC. The degree of between-studies statistical heterogeneity was evaluated with the use of the I^2 ^test for the diagnostic odds ratio [12]. The presence of a threshold effect on the diagnostic accuracy of pleural fluid sTREM-1 was evaluated with the Spearman correlation coefficient between the logits of sensitivity and specificity. We also performed meta-regression analyses in order to the effect of potentially confounding variables. The presence of publication bias was tested using funnel plots and the Egger test [13]. A p value < 0.05 was considered as indicative of statistical significance. All statistical analyses were performed using the following statistical software programs: Stata, version 10.0; Stata Corporation; College Station, TX; Meta-Disc for Windows; XI Cochrane Colloquium; Barcelona, Spain.

## Results

### 1. Description of studies

**Figure 1:**
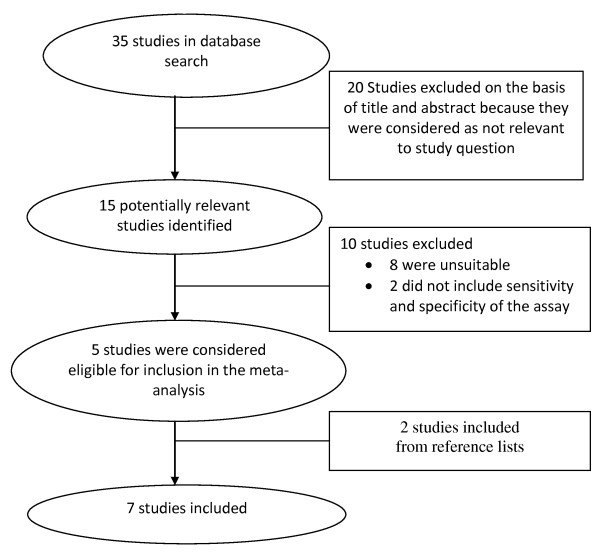
**Study selection process**.

The initial database search revealed 35 articles. From these 35 articles, 20 were excluded on the basis of title and abstract for being irrelevant to our study question, leaving 15 potentially relevant studies. Full articles of potentially eligible studies were reviewed in depth and finally 5 individual studies met our inclusion criteria [14-18]. 2 studies were further identified [19,20] from the reference lists of the 15 potentially relevant studies. Figure 1 shows the study selection process.

Table 1 and Table 2 summarize the main characteristics and results of the 7 included studies. A total of 733 patients were included in the meta-analysis. All the studies except one [17] were prospective studies. The means of diagnosis of bacterial pleural effusions, that is, the reference standard used in the studies included clinical and radiological findings, laboratory results, Gram-stain results, culture results and response to antibiotic therapy. 2 studies reported data for parapneumonic effusions only [19,20], 1 reported for empyemas only [16] and the rest reported for both parapneumonic effusions and empyemas. 2 studies [16,17] reported whether the assessment was done in a blinded manner, whereas that information was not reported in the other 5 studies. The cut-off values ranged from 50 pg/mL to 768.1 pg/mL. Enzyme-Linked Immunosorbent Assay (ELISA) was used for pleural fluid sTREM-1 measurements in all 7 studies. 1 study [14] also reported the measurement of surface TREM-1 by flow cytometry. Another study [15] reported concentrations of sTREM-1 in both pleural effusions and sera.

**Table 1:** Characteristics of studies included in the meta-analysis

STUDY/YEAR	COUNTRY	STUDY POPULATION	AGE	STUDY DESIGN	BLINDING OF INVESTIGATORS	MEANS OF DIAGNOSIS OF BACTERIAL EFFUSIONS	TYPE OF BACTERIAL INFECTION	ASSAY METHOD
Chan et al./2007	Taiwan	ICU/ hospital ward	NR	prospective	NR	clinical and biochemical findings; radiological studies; Gram stain; culture	10 PPE and 12 empyemas	ELISA

Kim et al./2007	Korea	Hospital ward	> 18	prospective	NR	clinical and biochemical findings; radiological studies; culture;response to antibiotic therapy	17 PPE	ELISA

Sim et al./2007	Korea	Hospital ward	NR	prospective	NR	clinical and biochemical findings; radiological studies; culture;response to antibiotic therapy	15 PPE	ELISA

Huang et al./2008	China	Hospital ward	17-81	prospective	NR	clinical and biochemical findings; radiological studies; culture;response to antibiotic therapy	4 PPE and 17 empyemas	ELISA

Bishara et al./2009	Israel	ICU/ hospital ward	NR	prospective	Yes	clinical and biochemical findings; Gram stain; culture	17 empyemas	ELISA

Porcel et al./2009	Spain	ICU/ hospital ward	NR	retrospective	Yes	clinical and biochemical findings; radiological studies; culture;response to antibiotic therapy	128 PPE and 30 empyemas	ELISA

Determann et al./2010	The Netherlands	ICU	NR	prospective	NR	clinical findings; Light's criteria; bacterial culture,response to antibotic therapy	5 PPE and 11 empyemas	ELISA

PPE: Parapneumonic effusions; ELISA: Enzyme linked immunosorbent assay

**Table 2:** Results of studies included in meta-analysis

STUDY/YEAR	NUMBER OF PATIENTS (n)	PREVALENCE OF BACTERIAL EFFUSIONS (%)	CUT-OFF (pg/mL)	TP	FP	FN	TN
Chan et al./2007	67	33	374	21	4	1	41

Kim et al./2007	48	35	55.4	12	8	5	23

Sim et al./2007	45	49	103.5	16	4	6	19

Huang et al./2008	109	19	768.1	18	6	3	82

Bishara et al./2009	89	19	114	16	5	1	67

Porcel et al./2009	308	50	80	115	42	43	108

Determann et al./2010	67	24	50	15	7	1	44

TP: True Positive; FP: False Positive; FN: False Negative; TN: True Negative.

### 2. Methodological quality of the included studies

**Figure 2:**
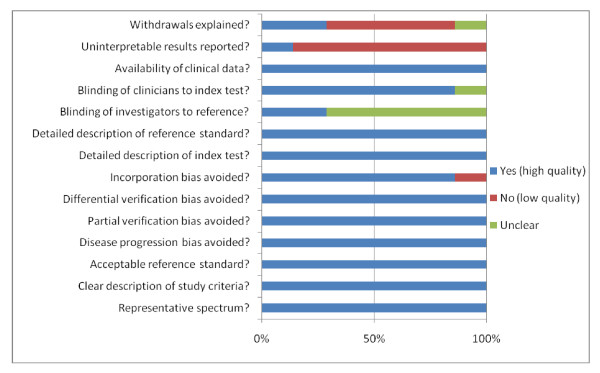
**Summary of the methodological quality assessment of the included studies**. Bars filled with blue/red/green color indicate the percentage of the studies that meet/do not meet the criteria/do not provide adequate relevant data, respectively.

In Figure 2, we summarize the findings of the methodologically quality assessment for the total of 7 studies included in our meta-analysis using the QUADAS tool. We attributed a score of 1 point for each "yes", 0 point for each "no" and 0.5 point for each "unclear". The maximum score using the QUADAS tool is 14.

The results of the methodological quality assessment of the studies included in the meta-analysis using the STARD checklist are shown in Table 3. The maximum score using the STARD checklist is 25.

**Table 3:** Summarized quality assessment using the STARD checklist

Section	Maximum score for each category	Study						
		**Chan et al.** [11]	**Kim et al. **[16]	**Sim et al. **[17]	**Huang et al. **[12]	**Bishara et al. **[13]	**Porcel et al. **[14]	**Determann et al.** [15]

Title and introduction	2	2	2	2	2	2	2	2

Methods	11	9	8	9	7	10	9	9

Results	11	5	5	5	8	7	6	8

Discussion	1	1	1	1	1	1	1	1

Total	25	17	16	17	18	20	18	20

### 3. Diagnostic accuracy of sTREM-1 for the detection of bacterial effusions

**Figure 3:**
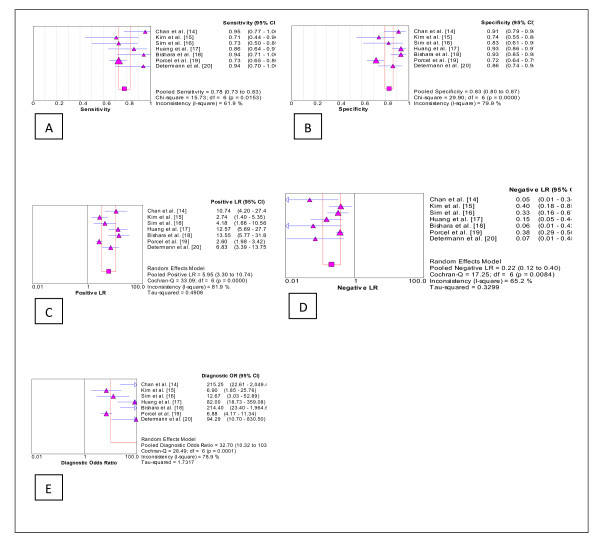
**Forest plots of *A*. sensitivity, *B*. specificity, *C*. positive likelihood ratio, *D*. negative likelihood ratio and *E*. diagnostic odds ratio of pleural fluid sTREM-1 for the diagnosis of bacterial effusions**.

The pooled (95% CI) sensitivity of pleural fluid sTREM-1 was 78% (72%-83%) and the pooled specificity was 84% (80%-87%). The pooled (95% CI) positive likelihood ratio was 6.0 (3.3-10.7), the negative likelihood ratio was 0.22 (0.12-0.40), and the diagnostic odds ratio was 33 (10-104). (Figure 3)

### 4. Threshold analysis and Summary Receiver-Operating Characteristics

**Figure 4:**
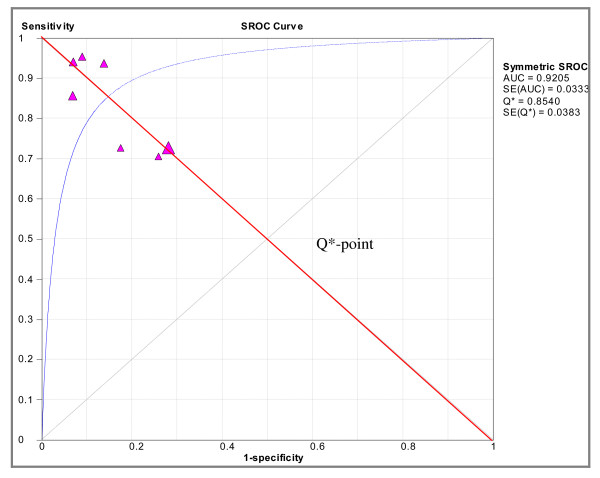
**Summary receiver operating characteristic (ROC) curve**. Observed values at study level are displayed (triangles) where y is the sensitivity and × is 1-specificity. The Q*-value sTREM-1 is 0.85 (SE 0.038).

As shown in Figure 4, a positive correlation was noted between sensitivities and 1-specificities. The Q* value, representing the highest common value of sensitivity and specificity was 0.854 (SE, 0.0383) and the area under the curve (AUC) was 0.92 (SE, 0.0333), indicating excellent diagnostic accuracy. No statistically significant difference was observed when exploring for threshold effect (n = 52, Spearman correlation coefficient = -0.643; p = 0.119).

### 5. Nomogram for likelihood ratios

**Figure 5:**
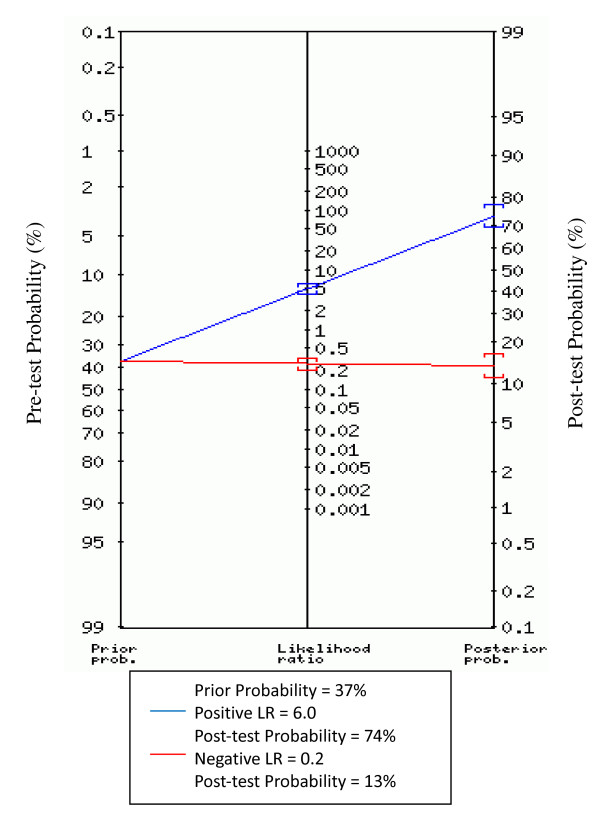
**Nomogram for likelihood ratios**. The nomogram shown is derived from the Fagan nomogram. The left side of the figure indicates a hypothetical pre-test probability of disease of 37%.

Figure 5 represents the nomogram for likelihood ratios, derived from the Fagan nomogram [21] and was generated by a web-based tool [22]. With a hypothetical pre-test probability of 37%, using pleural fluid sTREM-1 for the detection of bacterial effusion would raise the post-test probability to 74%. With a negative likelihood ratio of 0.2, using pleural fluid sTREM-1 reduces the post-test probability to 13%.

### 6. Investigation of heterogeneity

The Chi-square test was used to evaluate the presence of statistically significant heterogeneity across the studies and the inconsistency index (I-squared) was used to quantify the amount of heterogeneity. Statistical heterogeneity and inconsistency were found for sensitivity (p = 0.015, χ^2 ^= 15.73, I^2 ^= 61.9%), specificity (p = 0.000, χ^2 ^= 29.90, I^2 ^= 79.9%), positive LR (p = 0.000, χ^2 ^= 33.09, I^2 ^= 81.9%), negative LR (p = 0.008, χ^2 ^= 17.25, I^2 ^= 65.2%), and DOR (p = 0.000, χ^2 ^= 28.49, I^2 ^= 78.9%).

### 7. Meta-Regression analysis

We chose to investigate whether the QUADAS score, the STARD score, lack of blinding, and whether the studies were prospective or retrospective studies were responsible for the heterogeneity noted. The meta-regression analysis we performed showed that the QUADAS score (p = 0.3245; RDOR, 4.34; 95% CI, 0.11 to 164.01), the STARD score (p = 0.3331; RDOR, 1.70; 95% CI, 0.44 to 6.52), lack of blinding (p = 0.7439; RDOR, 0.60; 95% CI, 0.01 to 33.80), and whether the studies were prospective or retrospective studies (p = 0.2068; RDOR, 7.44; 95% CI, 0.18 to 301.17) did not affect the test accuracy.

### 8. Evaluation of publication bias

**Figure 6:**
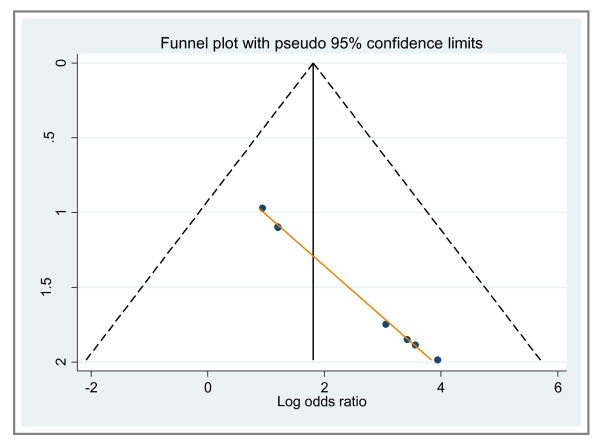
**Funnel plot for the assessment of potential publication bias in pleural fluid sTREM-1 for the diagnosis of bacterial effusions**. **(**The diagonal line represents Egger's line).

During the evaluation of publication bias, we found the p value for the Egger test to be statistically significant (p = 0.000). The funnel plot for publication bias (Figure 6) also suggests asymmetry, indicating a remarkable trend of publication bias.

## Discussion

Bacterial pleural effusions present a challenge for both diagnosis and treatment in many cases. Although significant recent advances have been made in the diagnosis and treatment of pleural infections, there still remains the need for further research if we are to reduce the morbidity and mortality caused by bacterial pleural effusions.

sTREM-1 is a diagnostic marker for sepsis and inflammation. It has been described as a diagnostic marker with a high accuracy and sensitivity in detecting microbial infections as underlying disease in critically ill patients [6,23,24]. The levels of sTREM-1 have previously been investigated in plasma, bronchoalveolar lavage fluid and exhaled breath [25-27]. In 2007, Liu et al. investigated this biomarker in pleural fluids for the first time and successfully demonstrated that it is a valuable marker in establishing the etiology of pleural effusions [28]. In the very same year, Chan et al. showed that pleural fluid sTREM-1 has a better diagnostic accuracy when compared to traditional parameters like WCC, neutrophil counts and LDH [14]. Since then, there have been several studies which have attempted to evaluate the diagnostic accuracy of this novel biomarker. In this regard, we performed this meta-analysis of 7 relevant studies in order to evaluate the accuracy of pleural fluid sTREM-1 for the diagnosis of bacterial pleural effusions. A total of 733 subjects were included in the study.

According to our findings, pleural fluid sTREM-1 seems to have a good diagnostic accuracy for bacterial pleural effusions since the area under the SROC curve was 0.92 and the Q* value was 0.85. The pooled sensitivity of pleural fluid sTREM-1 for the diagnosis of bacterial effusions was 78% and the specificity was 83%. Pooled positive LR was 5.95. With a hypothetical pre-test probability of 37%, using pleural fluid sTREM-1 for the detection of bacterial effusion would raise the post-test probability to 74%. With a negative likelihood ratio of 0.2, using pleural fluid sTREM-1 reduces the post-test probability to 13%, thereby showing that the application of pleural fluid sTREM-1 test to pleural effusions can be helpful in ruling out a bacterial pleural effusion.

An additional finding of our meta-analysis is that while the pooled DOR was 32.7, some studies achieved DOR as high as 215.2, while others attained a very low DOR of 6.9. The DOR is a single indicator of test accuracy [29]. The disparity noticed in our study can result from a number of reasons, for example, differences in the nature of the study population, whether they suffered from parapneumonic effusions or empyema. Since pleural fluid sTREM-1 levels are upregulated by the presence of endotoxins [30], we can stipulate that empyemas, having a higher burden of endotoxin will, most probably have a higher level of sTREM-1. This is confirmed by the sensitivity and specificity values of pleural fluid sTREM-1 in the studies included in our meta-analysis. Studies including empyemas only, for example, the study carried out by Bishara et al. [16] and those including a higher number of empyemas than PPE, that is, the ones carried out by Chan et al.[14], Huang et al. [15] and Determann et al. [18] attained higher DORs as well as higher sensitivities and specificities than those reporting pleural fluid sTREM-1 levels in PPE only or having a larger number of PPE than empyemas. The studies carried out by Determann et al. and Bishara et al. were the only two studies to provide details of the isolated pathogens from pleural effusions. However, they did not mention which organism cultured had the greatest impact on pleural fluid sTREM-1. Moreover, one patient from the study of Determann et al. and four patients from the study of Bishara et al. with macroscopic empyema had negative culture results and high pleural fluid sTREM-1 levels, thereby showing that even though organisms fail to be cultured, pleural fluid sTREM-1 is still high in pleural infection.

Although characteristics of patients and clinical settings were similar, and the same reference standards were adopted, there were marked differences in the size of the study populations and in the prevalence of bacterial effusions. Two studies reported higher prevalences as compared to others [17,20]. Porcel et al. carried out a retrospective study where they reported the samples to have been randomly chosen out of all stored samples from 2004 to 2008. The fact that not all bacterial pleural effusions and samples during that time period were used to calculate the prevalence might account for the high prevalence in this study. Sim et al., on the other hand, reported data for exudative effusions only and this is the reason for the high prevalence in this study.

Due to the fact that our meta-analysis revealed marked heterogeneity with regard to sensitivity, specificity, positive likelihood ratio, negative likelihood ratio and diagnostic odds ratio, we undertook a meta-regression analysis to explore the possible reasons for this heterogeneity [31]. However, none of the variables included in the meta-regression analysis were found to affect the diagnostic accuracy of pleural fluid sTREM-1. The 7 studies included in the study were considered to be of good quality, with the overall QUADAS score to be above 10 for all the studies and a score above 15 for all studies when using the STARD checklist. Despite the fact that lack of blinding also was found not to affect the diagnostic accuracy of pleural fluid sTREM-1 when we carried out the meta-regression analysis, we stipulated that heterogeneity might have partly attributed to false negative test results which might have gone undetected when there was no blinding. Additionally, the type of study included (prospective or retrospective) was not found to affect the diagnostic accuracy. This might be due to the fact that, out of the 7 included studies, only one was of retrospective nature. However, this one study accounted for the largest number of subjects included in the study, hence, a probable cause of heterogeneity.

The results of our meta-analysis are reliable due to the fact that even though the total number of studies included was small, the number of patients enrolled was satisfactory, hence decreasing Type II error. Additionally, sTREM-1 levels decline with therapy [32]. The use of antimicrobial therapy is likely to decrease the sensitivity of sTREM-1. The studies included in our meta-analysis reported sampling of pleural fluid for sTREM-1 analysis before the initiation of antimicrobial therapy. Our analysis had limitations that should be considered during interpretation of our findings. As mentioned earlier, most of the studies did not report blind testing and thus they were likely to overestimate accuracy, particularly sensitivity, thus underscoring the need to improve quality of design and reporting. Moreover, the lack of blinding might have also affected the overall sensitivity of the assay. It has been formerly proven that pleural fluid sTREM-1 levels differ in PPE and empyema [17]. Yet, we did not compute data separately for PPE and empyema due to the lack of data for each entity individually. For this same reason, we did not calculate a cut-off value for pleural fluid sTREM-1. Our meta-analysis, just like any other meta-analysis, could be influenced by publication bias as shown by the asymmetrical funnel plot in Figure 6[33,34]. Exclusion of case-reports, letters to editors and conference abstracts might have contributed to publication bias. Usually, studies having positive results have a greater tendency of being published and those with no results are usually not published, thus adding to publication bias in our analysis.

## Conclusions

In conclusion, according to our findings, sTREM-1 seems to be a reliable marker for bacterial pleural effusions since it has a good overall diagnostic performance. However, due to the fact that cut-off values vary greatly for parapneumonic effusions and empyemas, before sTREM-1 can be used widely in the clinical setting, more large blinded prospective studies must be carried out in parapneumonic effusions and empyemas separately to evaluate its accuracy.

## Abbreviations

DOR: Diagnostic odds ratio; LR: Likelihood ratio; SROC: Summary receiver operating characteristic; sTREM-1: Soluble triggering receptor expressed in myeloid cells-1

## Competing interests

The authors declare that they have no competing interests.

## Authors' contributions

HS contributed to literature search, review of articles, data analysis and drafting the manuscript. LLT contributed to literature search and review of articles. YGZ and HNJ contributed to reviewing of manuscripts and statistical analysis. JMQ contributed to literature search, data analysis and drafting of the manuscript. All authors read and approved the final manuscript.

## Pre-publication history

The pre-publication history for this paper can be accessed here:

http://www.biomedcentral.com/1471-2334/11/280/prepub
